# Challenges and Opportunities Developing Mathematical Models of Shared Pathogens of Domestic and Wild Animals

**DOI:** 10.3390/vetsci5040092

**Published:** 2018-10-30

**Authors:** Kathryn P. Huyvaert, Robin E. Russell, Kelly A. Patyk, Meggan E. Craft, Paul C. Cross, M. Graeme Garner, Michael K. Martin, Pauline Nol, Daniel P. Walsh

**Affiliations:** 1 Department of Fish, Wildlife, and Conservation Biology, Colorado State University, Fort Collins, CO 80523, USA; 2 U.S. Geological Survey, National Wildlife Health Center, Madison, WI 53711, USA; rerussell@usgs.gov (R.E.R.); dwalsh@usgs.gov (D.P.W.); 3 Center for Epidemiology and Animal Health, United States Department of Agriculture, Animal and Plant Health Inspection Service, Fort Collins, CO 80526, USA; Kelly.A.Patyk@aphis.usda.gov (K.A.P.); Pauline.Nol@aphis.usda.gov (P.N.); 4 Department of Veterinary Population Medicine, University of Minnesota, St. Paul, MN 55108, USA; craft@umn.edu; 5 U.S. Geological Survey, Northern Rocky Mountain Science Center, Bozeman, MT 59715, USA; pcross@usgs.gov; 6 European Commission for the Control of Foot-and-Mouth Disease—Food and Agriculture Organization of the United Nations, 00153 Roma RM, Italy; Graeme.Garner@fao.org; 7 Livestock Poultry Health Division, Clemson University, Columbia, SC 29224, USA; mmarti5@clemson.edu

**Keywords:** livestock, modeling, poultry, transmission, wildlife, wildlife-livestock diseases

## Abstract

Diseases that affect both wild and domestic animals can be particularly difficult to prevent, predict, mitigate, and control. Such multi-host diseases can have devastating economic impacts on domestic animal producers and can present significant challenges to wildlife populations, particularly for populations of conservation concern. Few mathematical models exist that capture the complexities of these multi-host pathogens, yet the development of such models would allow us to estimate and compare the potential effectiveness of management actions for mitigating or suppressing disease in wildlife and/or livestock host populations. We conducted a workshop in March 2014 to identify the challenges associated with developing models of pathogen transmission across the wildlife-livestock interface. The development of mathematical models of pathogen transmission at this interface is hampered by the difficulties associated with describing the host-pathogen systems, including: (1) the identity of wildlife hosts, their distributions, and movement patterns; (2) the pathogen transmission pathways between wildlife and domestic animals; (3) the effects of the disease and concomitant mitigation efforts on wild and domestic animal populations; and (4) barriers to communication between sectors. To promote the development of mathematical models of transmission at this interface, we recommend further integration of modern quantitative techniques and improvement of communication among wildlife biologists, mathematical modelers, veterinary medicine professionals, producers, and other stakeholders concerned with the consequences of pathogen transmission at this important, yet poorly understood, interface.

## 1. Background

Increasing human population growth, the subsequent increasing demand for food production (including increased meat and animal product consumption), and the conversion of natural habitats to agricultural land uses have all altered interactions between domestic and wild animal populations. Historically, transmissions of pathogens from livestock to wildlife have led to the maintenance of introduced livestock diseases in wildlife populations for up to a century or more [1], and vice versa. More recently, the loss and alteration of wildlife habitats due to anthropogenic activities have resulted in changes to pathogen and host distributions that provide increased opportunities for interaction between wildlife and livestock hosts, leading to pathogen spillover [2]. Spillover events from wildlife have resulted in the emergence of disease caused by previously unidentified pathogens, such as Hendra and Nipah viruses, and the resurgence of others, including avian influenza virus, African swine fever (ASF), and bovine tuberculosis (bTB) [2,3]. The increased likelihood of spillover events due to the proximity of wildlife and livestock contributes to the disease risk for both free ranging and domestic animals, as well as for humans.

Diseases affecting domestic animal populations are important from a variety of perspectives. For individual producers, disease-related morbidity and mortality of livestock negatively affects the production of animals and animal products, and, ultimately, may have animal welfare and financial implications [4,5,6]. From a regional or national perspective, livestock and poultry diseases can result in economic and trade consequences; for example, the 2001 outbreak of foot-and-mouth disease (FMD) in the UK led to the destruction of millions of animals and contributed to economic losses of over $9 billion USD [7,8]. Total costs of the 1997/1998 Netherlands Classical Swine Fever (CSF) outbreak have been estimated at $2.3 billion USD [9], and a total of $240 billion USD in economic losses have been reported as a result of the ASF outbreak in domestic and wild pigs in the Russian Federation between 2008 and 2011 [10]. For developing countries in particular, diseases of livestock and poultry can threaten food security and livelihoods [11,12].

Similarly, disease emergence, or re-emergence, among wildlife populations can negatively impact ecological systems and functions, as well as disrupt economic activities. Stakeholders such as hunters and wildlife-watchers can be affected by disease-mediated declines in wildlife populations with significant economic impacts; losses due to invasive animals and associated pathogens in the United States are estimated to cost $35 billion annually [13]. Spillover of pathogens from domestic animals to immunologically naïve wild animal populations can lead to drastic declines in wildlife populations, particularly if combined with other stressors, which have important conservation implications [14,15,16]. For example, the transmission of canine distemper virus from domestic dogs to wild African carnivores has been linked to population declines of some wild species [17,18,19]. Small populations that are already at risk are much more vulnerable to extinction in the face of disturbances such as the emergence of a novel pathogen [14,20,21]. Finally, disease can have indirect effects by altering the ecosystem’s functionality [22], resulting in impacts felt by many species beyond those directly affected.

Models are useful for predicting the effects of disease on populations, estimating the effects of management outcomes, and providing a framework within which trade-offs between different actions (including no action) can be evaluated [23,24]. However, unique challenges are posed when modeling wildlife diseases due to the relative paucity of information on wildlife compared to humans or domestic animals [23]. Specifically, host characteristics such as social structure, movement patterns, population numbers, and contact networks, which are vital aspects for capturing the potential spread of pathogens and the impacts of the emerging infectious diseases they cause [24,25,26,27], are more difficult to observe for wildlife species. Estimating the prevalence of infected or exposed individuals in a wildlife population is also often more difficult, requiring the capture and handling of animals or estimates from harvested animals which may not be reflective of exposure and infection rates in the non-harvested population [28]. Finally, although the need for approaches to disease management that draw together expertise from across disciplines—transdisciplinarity—is ever-increasing, a lack of effective communication and collaboration among various scientists and stakeholders, such as veterinary scientists, wildlife biologists, vector and microbiologists, statisticians, livestock owners, and managers, persists. This apparent continued segregation of disciplines exacerbates the difficulties of modeling diseases at the interface [29].

### 1.1. Disease at the Interface

A number of diseases have a shared component at the common boundary—or interface—between domestic and wild animal populations. The wildlife-livestock interface can be fundamentally defined as the continuum of direct and indirect contact between free-ranging wildlife and domestic livestock (or poultry). Indirect contact can occur through exposure to infected materials (such as aerosols, mucus, or feces) or through environmental reservoirs, such as soil water [30]. Overlapping habitats, including shared feeding grounds or watering points, can all provide opportunities for infectious pathogens to pass to and from domestic and wild animals. This interface is temporally and spatially dynamic, as the types and frequencies of interactions between wild and domestic animal species are influenced by daily, seasonal, and stochastic annual patterns in animal behavior and environmental conditions, as well as anthropogenic activities [31]. Habitat fragmentation, encroachment, and agricultural intensification, for example, provide greater opportunities for novel interspecific interactions. These interactions can include wildlife species that historically have not come in contact with each other, as well as contact between livestock and wildlife, which can lead to disease emergence [2,32]. For example, *Brucella abortus* transmission between domestic livestock and wild elk (*Cervus canadensis*) has occurred through contact with infectious birth materials at shared feeding sites [33,34,35], and Nipah and Hendra viruses emerged primarily due to land use changes that brought domestic livestock into greater contact with wildlife [36,37]. One Health approaches also recognize that humans are intrinsic to this continuum of contacts at the interface [1,16] because anthropogenic activities enhance the probability of novel interactions, intensify the outcome of interactions between livestock and free-ranging wildlife populations, and affect humans through the emergence of zoonotic pathogens.

### 1.2. Objectives

The complexities of the wildlife-livestock interface, the increased rate of the transfer of pathogens among wildlife, livestock, and humans, and the challenges associated with pathogen detection and disease management in wildlife, all necessitate a collaborative, transdisciplinary effort to develop novel, science-based methods to address disease concerns within these systems. The use of mathematical models is one such method and is increasingly recognized as a valuable tool for synthesizing information to better understand pathogen transmission routes and to support policies and programs aimed at the prevention and/or management of animal diseases [38]. In this context, transdisciplinary approaches are crucial to producing robust, powerful, and most importantly, useful multi-host dynamic transmission models. The objectives of this paper are to: (1) identify, qualitatively, the gaps and challenges in modeling pathogen transmission at the wildlife-livestock interface; (2) provide an overview of the quantitative methods and approaches to tackle these gaps; and (3) recommend a science-based path forward.

Considering the diversity of perspectives required to understand pathogen transmission at the wildlife-livestock interface, we approached our objectives by compiling ideas from both subject matter experts and the peer-reviewed literature. During a 2014 international workshop, we gathered a group of veterinarians, biologists, epidemiologists, statisticians, and mathematicians to evaluate the gaps and challenges in understanding and modeling pathogen transmission at the interface between free-ranging wildlife populations and livestock and poultry populations. A central focus of the group was to identify deficiencies in modeling transmission in livestock-wildlife disease systems using, as examples, FMD, bTB, highly pathogenic avian influenza (HPAI), and CSF; these diseases formed a foundation for discussion from which this broader paper emerged.

## 2. Importance of Mathematical Models of Disease Transmission at the Interface

Mathematical models are being applied with increasing frequency to improve our understanding of complex multi-host disease systems [38,39]. Models have been used to predict pathogen spread, to investigate disease control strategies, to develop risk analyses, and to study disease impacts on population dynamics [40,41,42,43]. A number of modeling approaches borrowed from population biology contribute to our knowledge of wildlife species occurrence in time and space and abundance, and can be used in the development of predictions regarding areas of overlap between wildlife and domestic animals during high risk pathogen transmission periods (e.g., brucellosis in elk and livestock [44]). In addition, mathematical models can help evaluate the contribution of proposed transmission pathways [45,46,47] to generating outbreaks, so that control efforts can be focused on the pathways that contribute the most to epidemics. Models can also assist by identifying the probability of pathogen eradication from livestock and/or wildlife under different management scenarios [6,48,49], identifying priority areas for surveillance [31,50], and predicting the likelihood of host extinction [21].

The development of predictive models of disease outbreaks caused by multi-host pathogens is hampered by the difficulty in determining: (1) the identity of hosts and pathogens, their distributions, and movement patterns; (2) the transmission pathways and rates between wildlife and domestic animals; (3) the effects of disease caused by pathogens and concomitant disease mitigation efforts on wildlife and livestock populations; and (4) barriers to communication among these sectors. Below, we explore these sources of uncertainty and gaps in our knowledge, as well as analytical approaches that have been used to deepen our understanding of disease in livestock, wildlife, or at the interface of both groups; a summary with some key examples is provided in Table 1.

### 2.1. Hosts and Pathogens: Their Distributions and Movement Patterns

Accurate identification of species that play a role in multi-host disease dynamics is an important and practical challenge. Identifying these species in wildlife disease systems is difficult and can often only be done by perturbing the system, for example, with an intervention technique and then intensively monitoring the system [74]. Our inability to identify all participants in the system can lead to the application of pathogen and disease control measures that are too generic to be effective, or even misapplied, in the event that the hosts, or geographic regions, being targeted are not actually those that are driving disease dynamics.

Once key species involved in the transmission and maintenance of pathogens are identified, we still often lack knowledge about their spatial and temporal distribution; information which is critical for predicting when and where disease might emerge. Systematic surveys for the presence of wildlife species can be logistically difficult and expensive, and such efforts are often constrained by limited available resources. Precise and accurate data on the locations of livestock hosts are not always available, either due to producer confidentiality and privacy concerns or due to a lack of infrastructure to obtain such information [75]. Distributions of pathogens in wildlife can also be difficult to estimate and are often only based on positive detections, without systematic surveys to confirm absence. For wildlife, species databases available to estimate the distribution of a disease can be hindered by low rates of detection of morbidity or mortality events because of limited observational opportunities, carcass loss or destruction, and underreporting, even where cases may be observed, particularly by the public [54]. If disease-associated morbidity and mortality make the detection of infected animals difficult, even systematic sampling efforts can result in biased estimates of infected individuals [76]. In addition, testing wildlife for diseases by collecting diagnostic samples often requires invasive capture methods or lethal sampling, which can be stressful for animals and researchers, and may give rise to public, legal, and animal welfare concerns [32].

Some surveillance programs, such as those for HPAIV and chronic wasting disease (CWD), have used hunter-killed samples to increase sample sizes at a low cost [77,78]; however, this method is limited to seasons and species that are subject to harvest. Non-lethal, non-invasive, and environmental sampling methods are actively being explored and/or implemented to enhance the sampling effort and frequency, while reducing the need for direct animal handling of wildlife. Camera traps to detect physical signs of disease [79], sampling feces to detect volatile organic compounds indicative of disease [80], the collection of saliva/oral fluids [81], and breath sampling [82] are all strategies currently being evaluated as noninvasive disease sampling tools.

Pathogen distributions in livestock hosts can be equally difficult to estimate. Underreporting may occur due to the infrequent observation of livestock and lead to delayed detection. Non-compliance in the reporting of notifiable diseases may also be an issue that can lead to underreporting of disease. Reporting of disease by livestock producers may be influenced by a number of factors, including an inability to recognize the disease [83], the potential deleterious impact of reporting disease on the individual farm through regulatory measures [84], and a lack of trust in the government [84,85].

The spatial or temporal resolution of the data may also influence our understanding of where and when a pathogen is present in a host population. For example, bats may be sampled for *Pseudogymnoascus destructans*, or Pd (the causative agent of white-nose syndrome), in the summer at roost locations to avoid disturbing hibernating colonies or due to a lack of knowledge regarding where winter hibernacula are located. Detecting Pd, however, is more difficult in the summer and it may be unknown where roosting bats overwinter [86]. Therefore, sampling at roost locations in the summer, though necessary in some circumstances, may lead to an incomplete picture of Pd presence on the landscape.

Most available population or host distribution data are incomplete or reflect imperfect detection [87]. Imperfect detection can have effects on inferences about pathogen and/or disease in both the wild and domestic animal components of multi-host systems if not properly accounted for. When modeling species distributions, however, the nature of the absence data (or the zeros) should be considered. Absence data in wildlife can be attributed to the true absence of the species, climatic or environmental conditions that prevent the species from occurring at the location, or methodological absences where no survey has taken place or the survey lacked methodological rigor [88,89]. Occupancy modeling [90] was developed for analyzing designed surveys of detection and non-detection to determine at which sites a species is truly absent versus undetected and is finding increasing utility in disease studies for both domestic and wild animal populations (Table 1).

Imperfect detection of pathogens may occur through the same mechanisms as imperfect detection of hosts; however, pathogens can also be subject to diagnostic testing bias. Tests used in wildlife are often only validated in domestic animals and are often not optimized for wildlife species. In the case of serologic tests, a positive result may only indicate a previous exposure or infection event and does not indicate an active infection [28]. Tests for diseases are rarely 100% perfect and, as with wildlife host species detection, multiple samples are often needed to accurately determine the presence or absence of the pathogen or disease [91].

Other techniques for the estimation of species distributions (including pathogens) are resource selection functions (RSFs) [92], generally applied to data sets consisting of multiple spatially referenced locations from individual animals (such as acquired from GPS collars), and niche modelling [55,93], often used for presence-only data and/or historical records. These techniques can be used to develop maps representing the potential habitat of wildlife and livestock hosts, allowing areas of overlap to be identified. However, if disease outbreaks are more closely tied to measures of abundance rather than estimates of presence, maps based on presence/absence data alone may be misleading [94], and other techniques such as spatio-temporal point process analyses [95] should be considered.

### 2.2. Transmission Routes, Rates, and Contact Networks

Modelling transmission at the interface between wild and domestic species is particularly challenging due to a lack of data on inter-species contacts, both direct and indirect, that might lead to pathogen transmission [45]. The force of infection (i.e., the rate at which susceptible animals become infected) is a key parameter in disease models and is particularly difficult to estimate in wildlife-livestock disease systems, or any multi-host system including humans, where partitioning the force of infection among the different host species is of interest [38]. The mode of transmission (i.e., direct contact, indirect contact, airborne, or vector-borne transmission) [96], as well as the nature and intensity of interactions between hosts, also influence transmission dynamics and are often unknown. Models of wildlife-livestock diseases focus on overlap and contact rates of wildlife and domestic hosts as key parameters driving the system [71,97,98,99,100]. However, identifying pathogen transmission pathways and contact rates between domestic and wild hosts is difficult [101]. Host species (domestic or wild) may be secretive or, in the case of environmental transmission, have left the area long before the disease emerges in a new host population. Models estimating contact networks between wildlife and domestic hosts often rely on range distribution maps, which are then overlaid. These methods ignore small-scale behaviors that may be important for pathogen transmission and often ignore a potential change in behavior due to infection [102]. Studies that explore these movements and contact patterns in the context of pathogen transmission, e.g., [103,104,105], have improved our understanding of transmission dynamics, although the results are likely specific to the characteristics of the study system (Table 1).

Identifying and describing indirect transmission routes such as the environment can be challenging for interface diseases. The environment can serve as a reservoir such that the indirect transmission of the pathogen to wild and domestic animal hosts may be an important and overlooked component of some disease ecosystems, including anthrax [53], low pathogenicity avian influenza (LPAI) viruses [106], toxoplasmosis [107], bTB [108], and brucellosis [44], among others. For example, *B. abortus* can be passed along from aborted bison fetal material on the landscape to cattle long after bison have vacated an area [33].

The traditional model of disease dynamics is the susceptible-infected-resistant model or SIR model [109,110]. Individuals transition between compartments based on the transmission rate of the pathogen between individuals and the length of the infectious period. The spread of disease in a population is controlled by the parameter *R*_0_ (the basic reproductive ratio), representing the average number of secondary cases caused by an infectious individual in a susceptible population. In terms of interface disease, SIR models have been used to examine the effects of alternative management scenarios on the risk of at least one bighorn sheep respiratory disease case resulting from contact with a domestic sheep [99] and to assess the risk of FMD transferring from saiga to livestock [71]. These models can be formulated in continuous time as ordinary differential equation models and have been widely used to represent disease dynamics (for a general overview see [39]; specific examples include [111,112] and Table 1).

Parameterizing SIR models can be difficult, for example, because capturing individual wild animals repeatedly to assess disease status, particularly animals that move large distances like migratory birds or bats, is often not possible. This can limit studies to a cross-sectional design which provides prevalence data for only a single point in time [28]. Estimating the rate of contact between individual hosts is also challenging, in large part because it is often difficult to define what a meaningful contact is (i.e., a contact that can potentially lead to pathogen transmission) and contacts are rarely observed (see [113] for a list of experimental techniques used to quantify contact networks in the field). In the majority of cases for animal diseases, we are left observing the outcome of transmission dynamics and inferring the transmission dynamics from models that replicate the observed outcomes, e.g., [114].

In general, SIR models assume homogeneous mixing of populations and are not spatially explicit. Network theory provides a method for describing the structure of social contacts in a population [27,113,115]. Using network theory, complex social behavior can be quantified and network graphs produced, representing the connectivity of the population. For example, some populations may consist of random infrequent contact between individuals, while other populations may be structured into family groups where contact rates are high within a group and low between groups. Network models can be used to explain why some populations may be more susceptible to disease than others and allow for an exploration of how the removal of particular individuals may differentially affect the spread of disease through a population [116]. Network approaches have been used for livestock [117] and have been increasingly used for wildlife disease systems [60,118] (Table 1).

Network models can also be used to inform agent-based models (ABMs) or individual-based models (IBMs), which are a type of spatially explicit model where agents (i.e., individuals) move within the landscape and interact with other agents according to a set of rules determined by the modeler [119,120]. Contact rates can be inferred from simulating populations and estimating the number of interactions from the simulated data, or defined by information from network models. ABMs allow for potentially more complex transmission dynamics, including the incorporation of environmental transmission, multiple hosts, animal movement behavior, and reservoir species. ABMs have been used to elucidate the transmission dynamics of feline immunodeficiency virus in bobcats [63], of a hypothetical microparasite in red colobus monkeys [62], and plague in prairie dogs [121], but may be more difficult for multi-host systems (Table 1).

Realistic parameterizations of some model components of wildlife-livestock disease dynamics may be possible using population genetics approaches. Examining the genetics of microbes and pathogens is a promising avenue forward for identifying potential transmission pathways between species [122]. For example, whole-genome sequencing of *B. abortus*, the causative agent of brucellosis, revealed that the pathogen was historically introduced to wildlife on at least five occasions in the Greater Yellowstone Ecosystem, but that contemporary livestock cases were coming from elk [35]. The genetics of *Escherichia coli* have been used to establish social contact patterns in giraffe (*Giraffe camelopardis*) [123] and potential pathogen transmission pathways between mongoose (*Mungos mungo*) and humans [124]. Cowled et al. [125] studied *Salmonella* infection and risk factors in a wild pig population using genetic methods and Blackburn et al. [126] used genetics of *Bacillus anthracis* to verify the role of blowflies in the transmission cycle of *B. anthracis*. Other studies have combined pathogen genetic and epidemiological information to estimate transmission trees for an avian influenza outbreak among poultry farms in the Netherlands [127] and FMD outbreaks in the UK [128].

Finally, though most modeling efforts focus on one disease-one host systems, when evaluating the impacts of disease and mitigation strategies, it is important to consider that disease systems may include multiple hosts [129]. The dynamics of diseases that include multiple-hosts are inherently more complex. For example, it may be difficult to identify hosts for a disease that kills species A but not species B, and species B is able to transmit the pathogen a long distance to a new population of species A [121]. Multiple disease agents may also impact the disease dynamics with infections from one pathogen repressing or enhancing the ability of a second pathogen to establish itself in a host species [39,130,131,132].

### 2.3. Modeling the Effects of Disease and Mitigation Strategies

Estimating the impacts of disease on host populations at several levels can also be biased by imperfect detectability. Diseased individuals may hide, become isolated from healthy individuals, be depredated or scavenged, or die before detection and confirmation of the individual’s disease state. These are all scenarios under which the impact of disease on a population (i.e., survival, reproduction) would be underestimated [87]. Estimates of population-level effects will be biased when sampling schemes do not take variable detection in space or time into account (e.g., convenience sampling) [133,134]. Accounting for imperfect detection at other levels, such as in the estimation of total host population size before or after the disease introduction [87], will also be important for accurately assessing the impact that disease has on host populations.

Evaluating and modelling population-level impacts of disease may be complicated by genetic variability among hosts or pathogens. Comparisons among studies may lead to different conclusions regarding the lethality of the disease if different types of host or strains of pathogen are grouped together. For example, some strains of pathogen may be more virulent than others [135], while genetic variability among hosts may mediate the responses to disease for particular genotypes [136]. Variability among hosts can potentially be exploited to promote disease mitigation, for example, management techniques for eradicating bTB from Europe and elsewhere have shifted the focus of control efforts towards approaches that harness the genetic variation in the host response to infection [137]. These techniques rely on the ability of the host to adapt more quickly than the pathogen can evolve into a new strain. Strain variation of pathogens, for example, avian influenza viruses, makes predicting the effects of future disease outbreaks on host populations difficult, in large part because of the complexities associated with accurately predicting which strain or strains will emerge next [138].

For wildlife species, detecting the effects of fast-acting and highly pathogenic diseases can be difficult if individual sampling is intermittent (e.g., canine distemper virus in carnivores). Individuals are likely to be infected and then die prior to being tested (or re-tested), and, as such, may be misclassified as uninfected prior to death. In many wildlife cases, disease may be a predisposing factor reducing survival, but the proximate cause of death may be interspecific competition, road kill, predation, starvation, or co-infection, etc., such that evaluating the impacts of disease on host populations may require experimental manipulation of the system to demonstrate whether disease regulates a host population [139]. In the absence of such comprehensive experiments, one approach has been to evaluate changes in components of fitness, such as survival and reproduction, such that a population-level impact of disease in free-ranging animals is reported as a decline in survival (or an increase in its complement, mortality) or a decline in some measure of reproductive output [140]. Singly or in combination, declines in these measures of components of fitness can serve as indicators of population-level impacts of disease on hosts.

An essential element for developing a mitigation plan is a model of how the system “works”. At a minimum, a host-pathogen model requires the integration of knowledge regarding the spatial distribution of the disease agent and the hosts, the transmission dynamics of the pathogen causing the disease, and the changes in demographic rates and/or behavior associated with infection [25,97,141]. Once a model that is reflective of the host(s), pathogen(s), and the environmental milieu they share is developed, the long-term impacts of disease on a population and mitigation or control efforts can be simulated to identify where or when disease management may be most effective (Table 1) [142,143,144]. A suite of potential mitigation measures may be used to control pathogens at the wildlife-livestock interface, including, for example, depopulation or population reduction, vaccination, vector control, containment, sterilization, or therapeutics. The choice of mitigation measure(s) in the event of an outbreak depends on many factors, including, but not limited to, pathogen and/or transmission characteristics, the severity of morbidity and mortality among hosts, host species infected or at risk of infection, available technologies, and the level of political/trade/economic implications. In addition, choice of mitigation strategy is further influenced by potentially competing interests from human health, agricultural/domestic animal health, and conservation perspectives [15].

Both field- and laboratory-based experiments can be used to inform models by providing estimates for these important parameters, though effective wildlife experiments can be challenging to accomplish. For laboratory experiments, some pathogens can only be manipulated under strict conditions, animals can be difficult to obtain, and maintaining populations of wildlife species under laboratory conditions can require new animal husbandry procedures [76]. In addition, extrapolating the results of experiments conducted under laboratory conditions to field conditions can be tenuous because natural conditions may vary substantially in terms of animal densities, types of animal contacts and frequencies of contact, or climatic conditions [145,146].

Applying disease mitigation strategies in the field and conducting field-based experiments can be difficult (due to the stochastic events such as fire or drought which can eliminate experimental animals), ethical concerns that result in small samples sizes or lack of replication among study sites, and difficulty in controlling animal movements between control and treatment sites [147]. Finally, an inability to trap and capture, or remotely target, sufficient numbers of the host species for the administration of a treatment or for culling can be an impediment to successful mitigation. For example, Pederson and Fenton [148] estimated the percentage of the population that should be treated with anti-parasitic compounds in order to have population-level effects, but this number may be untenable for wildlife species. Despite these difficulties, controlled experimental design studies in the field and laboratory offer great promise for parameterizing disease models designed to predict the effects of mitigation strategies on host and pathogen populations [45].

Decision theory offers an established framework for evaluating the trade-offs between potential mitigation actions, determining which factors in the system under consideration are most likely to affect the outcome of the model and/or the optimal decision, and, most importantly, accounting for the societal values associated with different predicted outcomes [149]. Decision theory can allow for the incorporation of information from the modeling techniques previously described in this paper to develop a model of “how the system works” [150]. This model can then be perturbed to estimate the effects of a management tactic or stressor, such as disease, on the system. By conducting sensitivity analyses on host-pathogen models, the most important parameters can be identified and experiments can be designed to reduce the uncertainty surrounding that particular parameter [151]. For example, for vaccination strategies, important parameters may be vaccine efficacy, the duration of vaccine-derived immunity, and optimal timing of doses or the need for boosters [145,152,153]. Iterative decisions can be evaluated using an Adaptive Management framework to distinguish among potential hypotheses [154].

### 2.4. Effective Communication

Effective communication across disciplines is critical for bringing together different data streams and different perspectives on wildlife-livestock diseases, which enhances our understanding of the processes that lead to the emergence of disease and the ways to mitigate the effects of disease. Data sharing, along with consistent methodologies and protocols that allow comparisons among data sets, development and use of a common lexicon, and incentivization of transdisciplinary collaborations are necessary for effective communication to occur. Data sharing is an essential aspect of fostering research that crosses the boundaries of traditional disciplines. In order to share data, standards of data management must be adopted by practitioners and researchers [155], and data analysts and technicians with the skills to manage data properly and proactively (rather than after the data have been collected) should be included when planning any data collection activity. Hindrances to data sharing include fears that data will be misused or not properly credited. In some cases, there may be privacy or confidentiality issues that may need to be managed through data sharing agreements. The ability and willingness of researchers to share data in a responsible fashion could be included as part of funding decisions [155] to incentivize the practice; a number of funding agencies and publication outlets require data sharing in public, electronic repositories.

In addition to data management practices, standard protocols for the collection of pathogen and disease data, and quality control procedures, such as proficiency testing, sending samples to different laboratories to be tested [156], and using recognized reference laboratories, should all be regular practices, particularly in the early stages of an outbreak when the identification and diagnosis of new diseases are critical. A registry of validated tests for animal diseases is available from the World Organization for Animal Health (http://www.oie.int/our-scientific-expertise/certification-of-diagnostic-tests/the-register-of-diagnostic-tests/) and standards and guidelines for diagnostic tests are provided (http://www.oie.int/international-standard-setting/terrestrial-manual/access-online/). Adherence to these guidelines should be encouraged, and perhaps required, for publication, to make comparisons among studies possible. At the same time, these tests are typically only fully validated in livestock; a consensus among collaborators regarding which diagnostic techniques and metrics will be used in studies involving wildlife disease should be formed in the initial stages of a project to encourage the deepest level of understanding about the disease system.

In addition to standard protocols, the establishment of a common lexicon for describing disease promotes and enhances effective communication among disciplines. For example, case definitions (such as that for *Batrachochytrium salamandrivorans*) [157] ensure that criteria for a definitive diagnosis are consistent between studies and assure that terms are used consistently. As important, but perhaps more difficult than determining criteria for diagnosis, is the establishment of a common lexicon for the meaning of fundamental words about disease systems, such as “outbreak”, “exposure”, “risk”, and “health”. Patyk et al. [158], for example, describe how the definitions of health may vary between veterinarians and wildlife biologists for the polar bear (*Ursus maritimus*) and how the establishment of a distinct definition of health can improve the management for this species. Practices such as those outlined in Patyk et al. [158] will lead to improved communication among disciplines by providing definitions of abstract concepts that practitioners can agree upon.

Finally, although there is general agreement that transdisciplinary collaboration is necessary to improve our understanding of diseases at the wildlife-livestock interface, several impediments to true transdisciplinarity have been identified. These impediments include a lack of funding for research among disciplines [159], a lack of cross-referencing of publications between journals in different disciplines [29], and skepticism toward the credibility of others and their work outside of one’s own discipline [160]. The incentivization of transdisciplinary work can occur through the promotion and maintenance of programs that support such work, including the NIMBioS workshop that fostered our initial discussion (http://www.nimbios.org/), as well as other organizations that promote and fund work that draws multiple disciplines together for disease research (Marie Skłodowska-Curie Actions, https://ec.europa.eu/research/mariecurieactions/; National Science Foundation, EEID program, https://www.nsf.gov/). Participation in collaborative work, especially when a researcher may be one of many collaborators across an array of disciplines, is gaining increasing recognition by—and inclusion in—promotion and tenure processes in academia [161]. In summary, effective communication is key to overcoming the challenges to modeling diseases at the wildlife-livestock interface that we have highlighted. To move forward together, and to learn the most about interface disease systems, will necessarily require improved data sharing, standardization of protocols and data management practices, and, perhaps most importantly, overcoming misperceptions about work that spans multiple disciplines.

## 3. Conclusions

Modeling diseases across the wildlife-livestock interface involves many challenges. We have identified four key components necessary for effectively modeling disease at the wildlife-livestock interface: (1) host and pathogen distributions and movement patterns, (2) transmission rates and pathways, (3) estimates of disease effects, and (4) effective communication, and we have summarized the challenges associated with describing these components for wildlife-livestock diseases (Table 1). Management of diseases at the wildlife-livestock interface requires input from both the domestic animal and wildlife sectors, and it necessitates that management activities be carried out among livestock/poultry populations, among wildlife, and at the interfaces between them.

The development of a model to describe how the system works, whether conceptual or mathematical, is a crucial step for managing shared diseases. Models can help with predicting long-term outcomes of disease, informing trade-offs between different management strategies, estimating the potential effects of mitigation, and identifying key parameters in the system where further research is necessary. Management of diseases at the interface should make considerations for evaluating long-term success, impacts to stakeholders, cost, species and conservation impacts, and ecological consequences [162,163]. Models provide a framework for addressing all of the above [164] when making decisions regarding disease management and we recommend the development of models as a critical step for fully understanding disease processes. We also suggest that efforts that focus on resolving uncertainty in key parameters of the disease system under study that we have highlighted here (i.e., host and pathogen distributions, movement and contact networks, and transmission dynamics) will garner significant benefit to the management of wildlife-livestock interface disease problems. Furthermore, substantial gains can be made by integrating both the modeler and field-based practitioner in a collaborative, iterative research framework [165].

Communication among disciplines is key to successfully modelling diseases at the wildlife-livestock interface. Ecologists, veterinarians, economists, computer programmers, policy advisers, disease specialists (e.g., virologists, microbiologists, mycologists, vector biologists), agricultural specialists, wildlife managers, emergency planners, social scientists, modelers, statisticians, and mathematicians, among others, will all have valuable perspectives to contribute and their perspectives should be engaged in the earliest stages of disease emergence. Incentives for collaborative work, increased opportunities for professionals to develop working relationships with those in other fields, recognition of the scientific advancements that a transdisciplinary approach can provide, and the intrinsic value of working as part of a team will all be necessary for the successful management of these diseases.

## Figures and Tables

**Table 1:** Three central sources of uncertainty and outstanding questions (‘knowledge gaps’) encountered when considering mathematical models of disease transmission at the livestock-wildlife interface.

Sources of Uncertainty	Key Knowledge Gaps	Analytical Approaches	Literature Examples
*Distribution and movements of hosts and pathogens*	When and where do livestock and wildlife hosts overlap?	Resource selection functions	Elk and brucellosis [44]
			Deer and chronic wasting disease [51]
	Is the geographic range of the pathogen the same as that of the host(s) or is the pathogen constrained by environmental conditions?	Presence-only models	Anthrax distribution [52]
			Plague distribution [53]
			Ecological niche modeling in general [54]
	When and where does pathogen exposure result in population extinction?	Occupancy models	Chagas disease vectors [55]
			Chytrid fungus and frogs [56]
*Transmission pathways and rates*	How do animals become infected? Direct contact? Indirect contact?	SIR models	Multiple species and rabies [57]
			Pigs and influenza A [6]
	What are the most important pathways for transmission between wildlife and livestock?	Contact networks (with SIR models)	Lions and distemper [58]
			Parasite transmission [59]
			Raccoon rabies [60]
	Are there features in the landscape that facilitate or prevent the spread of the pathogen?	Agent-based models	Colobus monkeys [61]
			Prairie dogs and plague, individual model to simulate SIR dynamics [46]
			Bobcats and FIV [62]
		Diffusion models	Feral swine and FMD [63]
			Raccoon rabies [64]
			Chronic wasting disease and deer [65]
		Metapopulation models	Raccoon metapopulations and rabies [66]
			Raccoon and skunk rabies [67]
*Effects of disease and mitigation on host populations*	What long-term impact does disease have on the host population?	Population viability models	Seabirds and avian cholera [68]
	Where are the high-risk areas to target mitigation efforts? What scenarios lead to greater risks of transmission?	Optimization	Raccoon rabies and bait distribution [66]
			Bovine tuberculosis [69]
		Risk assessment	Many examples including:
			Salamanders and chytrid [50]
			Raccoon and skunk rabies [67]
			Saiga antelope, livestock, foot and mouth disease [70]
	What are the trade-offs among alternative mitigation strategies? What are likely to be the most effective mitigation techniques?	Decision theory	Few examples but see: Cost-benefit of wildlife-livestock disease mitigation [71]
		Multiple scenario risk assessment	Bison and brucellosis, alternative management actions [72]
			Plague and prairie dogs, alternative climate scenarios [73]
